# Markerless Radio Frequency Indoor Monitoring for Telemedicine: Gait Analysis, Indoor Positioning, Fall Detection, Tremor Analysis, Vital Signs and Sleep Monitoring

**DOI:** 10.3390/s22218486

**Published:** 2022-11-04

**Authors:** Lazzaro di Biase, Pasquale Maria Pecoraro, Giovanni Pecoraro, Maria Letizia Caminiti, Vincenzo Di Lazzaro

**Affiliations:** 1 Research Unit of Neurology, Neurophysiology and Neurobiology, Department of Medicine and Surgery, Università Campus Bio-Medico di Roma, Via Alvaro del Portillo 21, 00128 Rome, Italy; 2 Operative Research Unit of Neurology, Fondazione Policlinico Universitario Campus Bio-Medico, Via Alvaro del Portillo 200, 00128 Rome, Italy; 3 Brain Innovations Lab, Università Campus Bio-Medico di Roma, Via Álvaro del Portillo 21, 00128 Rome, Italy; 4 Department of Electronics Engineering, University of Rome Tor Vergata, 00133 Rome, Italy

**Keywords:** gait analysis, indoor positioning, fall detection, tremor analysis, vital signs monitoring, sleep monitoring, telemedicine

## Abstract

Quantitative indoor monitoring, in a low-invasive and accurate way, is still an unmet need in clinical practice. Indoor environments are more challenging than outdoor environments, and are where patients experience difficulty in performing activities of daily living (ADLs). In line with the recent trends of telemedicine, there is an ongoing positive impulse in moving medical assistance and management from hospitals to home settings. Different technologies have been proposed for indoor monitoring over the past decades, with different degrees of invasiveness, complexity, and capabilities in full-body monitoring. The major classes of devices proposed are inertial-based sensors (IMU), vision-based devices, and geomagnetic and radiofrequency (RF) based sensors. In recent years, among all available technologies, there has been an increasing interest in using RF-based technology because it can provide a more accurate and reliable method of tracking patients’ movements compared to other methods, such as camera-based systems or wearable sensors. Indeed, RF technology compared to the other two techniques has higher compliance, low energy consumption, does not need to be worn, is less susceptible to noise, is not affected by lighting or other physical obstacles, has a high temporal resolution without a limited angle of view, and fewer privacy issues. The aim of the present narrative review was to describe the potential applications of RF-based indoor monitoring techniques and highlight their differences compared to other monitoring technologies.

## 1. Global Overview on Current Monitoring Systems

In this narrative review, we describe, as a main aim, the potential applications of RF-based indoor monitoring techniques and highlight differences with other monitoring technologies. In order to provide an overview on the topic, we first describe the features of the most-used existing technologies for outdoor and indoor monitoring, compare their performance and finally explore their clinical applications for gait analysis, fall detection, tremor assessment, vital signs, and sleep monitoring.

The objective quantitative and continuous assessment of motor performances and the monitoring of vital signs are currently a crucial issue not only for professionals but also for patients as it could improve clinical practice by providing a careful follow-up and a more tailored therapy. Recent trends in the field of personalized medicine highlighted the necessity of empowering medicine with available telemonitoring technologies [1]. Good example are given by movement disorders like Parkinson’s disease [2,3,4,5,6,7,8], dystonia [9,10,11] or tremor [12,13,14,15], in which telemonitoring solutions are a promising approach to improve the diagnostic and therapeutic process [11,16]. This approach has been further motivated by the upcoming requests of patients suffering from chronic disorders and their caregivers to receive customized therapies with high accuracy standards and at low costs, since indoor activities appear to be globally more demanding for older people for various reasons. First, indoor environments can be narrow or wet and generally complicated by the presence of objects or moving people: sometimes, even a simple walk in such a highly constrained scenario can increase the risk of a fall for an old-aged subject. Second, the activities of daily living (ADLs) frequently require more fine and precise actions, e.g., cleaning, cooking, or dressing, than outdoor activities. Finally, time spent indoors is generally preferred by frail patients, who tend to consider these locations as “safer”. Taking into account these facts, focusing on indoor activities monitoring is a promising prospect for patients. Furthermore, over the last years, a positive impulse for medical assistance has been achieved by the introduction of contactless clinical assessment systems, with the possibility for the patient to be visited not only in the hospital but also in an at-home setting, consistent with recent trends of telemedicine. In the context of a progressively older and chronic-illness-affected population, telemedicine has been challenged to improve the efficacy of existing human activity monitoring models, e.g., to prevent acute worsening and falls. It is worth noting this is totally in line with the wide expansion of smart homes that offer the possibility to connect sensors and smart devices to a centralized network where the recorded data can also be used to train machine learning models. Recognition of activity patterns in smart homes has demonstrated promising results [17]. Moreover, as highlighted in [18,19], changes in the amount of global activity, percentage of time spent in different rooms and type of activity performed by a subject can be an early indirect sign of neurodegenerative disorders such as dementia. Major classes of devices proposed for movement detection are inertial, optical, geomagnetic-based and radar sensors. 

### 1.1. Inertial Monitoring

For outdoor activities wearable devices offer better performances since they can follow the subject in any location [20]. Instead, camera and RF-based devices have limited application outdoors due to their limited range of detection, which is strictly dependent on the distance/angle from the sensor. Inertial-based devices have been widely investigated in the literature [21]: they can estimate the position of a person by combining data from accelerometers [22], gyroscopes [23], acoustic sensors [24], and even from smartphones [25,26]. Furthermore, they globally share the same mechanical principle, i.e., they need to be worn or be in contact with the subject’s body. Their features allow them an extensive range: high accuracy, low costs, light-weight size, little hindrance to natural movement and long-term monitoring [27]. In addition, recent studies have highlighted the potential role of integrating machine learning algorithms with data collected from these sensors as a diagnostic tool, especially in the movement disorder clinic [28,29,30,31,32,33]. Machine learning models can achieve high classification accuracy since they can “learn” from large-scale datasets by extracting features, such as kinematic parameters of gait [31,32,33], tremor [28], and pattern recognition. In particular, unsupervised methods are generally preferred, due to their autonomous learning without any human support [34]. However, some drawbacks are intrinsically related to the properties these devices are based on. First, the correct position of the tool on the patient’s body is required for a high-quality recording of kinematic parameters. In addition, once placed on a specific body part, the recordings are limited to that site [35]. The battery must be periodically charged, and often replaced with a new one. Researchers tried to solve this problem with the introduction of discrete instrumented insoles based on pressure sensors, which maintain good accuracy standards with low energy consumption, but the insertion of this additional insole in a shoe limited their usability [36]. Finally, monitoring in more dangerous scenarios, such as in bathrooms, could be very difficult, since the usage of wearable solutions is not always viable, e.g., during a shower.

### 1.2. Camera-Based Monitoring

Camera-based solutions outperformed the limitations of wearable sensors with regard to invasiveness as strict contact with the body is not needed [18,37,38]. This kind of monitoring system takes advantage of depth data with the possibility to decompose movement in its constitutive elements [18,39]. Vision-based devices are generally integrated with deep learning algorithms that process acquired data, that belong to different classes, usually organized in fall or non-fall classes [18]. However, limited angle of view, barriers, light conditions, and necessity of colored markers on the subject’s body represent major drawbacks. Last but not least, camera-based devices raise major privacy concerns which are quite critical, since monitored people and their living environments are completely depicted. Despite the aforementioned pioneer investigations in the literature, the described indoor localization and motion-tracking models appear inadequate to guarantee a continuous and non-invasive monitoring. 

### 1.3. Radio Frequency Monitoring

**Figure 1:**
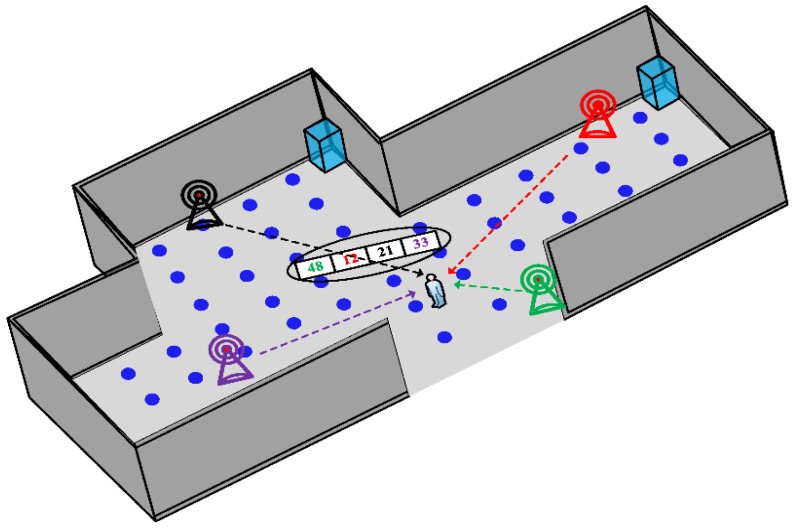
The working process of Wi-Fi fingerprinting indoor localization. A comparison of collected fingerprints in the database helps to estimate the locations of different clients (reproduced under the terms and conditions of the Creative Commons Attribution (CC BY) license from [43]).

Researchers had to re-think the paradigm of movement monitoring by considering alternative models that had to simultaneously not interfere with the clinical visit and not to be affected by physical obstacles, e.g., people, clothes, and walls. New standards in indoor assessment should focus on the introduction of user-friendly contactless equipment as light and small as possible, providing discrete monitoring under a natural environment. It is mandatory to think about alternative solutions of non-invasive tracking which can also preserve a good measurement quality. The pervasiveness of the new communication standards on people’s everyday life and the recent reconfiguration of radar architectures have generated growing interest in the medical community for radio-frequency (RF) technologies. RF-based techniques represent a novel and heterogeneous category of indoor localization systems, whose physical principle takes advantage of both wave emission and interaction between waves and surfaces encountered to detect objects in the space. RF-based systems are not dependent on lighting [18,38,40], less affected by physical hindrances [41,42], have poor dependence on subject compliance and are also characterized by low energy consumption. For this purpose, currently used techniques include wireless communication systems, such as Wi-Fi, Long-Term Evolution (LTE), Bluetooth, Radio Frequency Identification (RFID) and Ultra-Wide Band (UWB) [43]. Easy access and wide availability make Wi-Fi a viable fingerprinting-based method. Wi-Fi-based indoor localization is feasible due to non-line-of-sight (NLoS), low cost, high reliability and wide availability of Wi-Fi-enabled devices [43]. In Figure 1 the working process of Wi-Fi fingerprinting-based method is shown. He et al. [44] showed that Wi-Fi fingerprinting, without knowing the exact access points (APs) locations, can achieve high feasibility in indoor positioning. Furthermore, a mobile application has been proposed for the estimation of the position of a user within a building by using Wi-Fi technology [45]. However, more complex large-scale positioning suffers from many other interferences [46]. In addition, it has been demonstrated that Wi-Fi integrated with machine learning algorithms can determine a building’s occupancy profile: in an on-site experiment an occupancy prediction model using Wi-Fi probes showed accuracies between 80% and 93.9% [47]. RF fingerprinting has also exploited the LTE signals already available in an environment not only for indoor [48,49,50,51] but also for outdoor [48] localization since they are transmitted by public base stations.

The RFID positioning system consists of readers and tag devices. Multiple infrared sensors placed in an indoor environment are exploited to estimate the distance and angle of the signal source. Relatively good accuracy standards are obtained for empty indoor environments, but drawbacks are related to the complexity of the model and the marked susceptibility to physical obstacles [43]. In the review by Li and Becerik-Gerber [52], the major location-sensing methods used in RFID-based solutions are summarized, deepening the RFID-based indoor location-sensing (ILS). The authors stated that no single solution satisfies all criteria for widespread validation [52]. 

Bluetooth Low Energy (BLE) technology raised attention for its availability in smartphone devices, low cost, and power demand [53]. BLE positioning uses information from beacons installed in a building and has found application for remote healthcare monitoring [54,55], indoor navigation [56] and activity recognition [57]. Occupancy estimation in an indoor environment can be obtained only with BLE beacons, a mobile application, and a server. In the experimental models of [Filippoupolitis, Oliff [53,58]], BLE integrated with three machine learning classifiers (k-nearest neighbors, logistic regression and support vector machine) was tested to unveil the presence of subjects inside an office, achieving promising results for occupancy estimation. A network of BLE beacons was further used with a machine learning model to study set of occupancy profiles and patterns of an area [59]. In a study by Tekler, Low [60], a two-week data collection was conducted to identify occupation patterns in a university office environment. A non-intrusive occupancy monitoring approach based on pre-existing BLE in smartphones was applied to track the occupants’ movement patterns, without any application installation.

**Figure 2:**
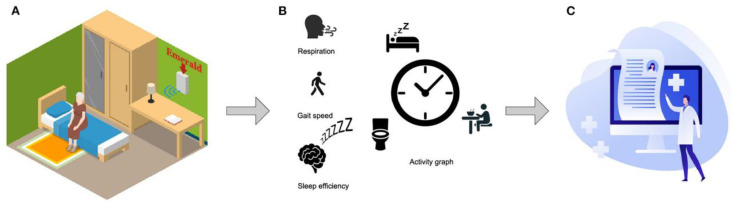
The Emerald RF sensor. Picture (**A**) shows the analogy with a Wi-Fi router. in the picture (**B**), recording of respiration, gait speed, sleep efficiency and daily activities patterns are collected. Finally, the picture (**C**) exhibits the possibility for qualified clinicians to remote acquire the recorded data (Reproduced under the terms and conditions of the Creative Commons Attribution (CC BY) license from [61]).

A fascinating proposal is to focus radar-based localization systems on gait analysis, vital signs, sleep monitoring, and fall prevention for older patients, especially in indoor settings. In Figure 2, the Emerald sensor is presented [61]. It has been validated for different clinical settings: it operates by transmitting very low power wireless signals and infers respiratory signals, gait speed, sleep patterns and time spent in different locations at home (activity graph) by the analysis of signal reflections due to human and inanimate objects [62,63]. It can also collect data continuously for prolonged periods, without any burden for patients or caregivers (see Figure 2).

In particular, Impulse-Radio Ultra-Wideband (IR-UWB) indoor monitoring systems are based on radar technology and have been demonstrated to be accurate and reliable in a variety of indoor environments [64], while the reliability is poor for outdoor monitoring. Furthermore, precision standards are generally lower than inertial-based sensors. Differently from the camera-based approaches, the monitoring of a single individual movement over a group of people represents a big challenge for RF systems. The usage of antenna arrays [65] or Multiple-Input Multiple-Output (MIMO) [66] techniques represent the most common solution to isolate signals from multiple subjects. Another example is given by the breathing separation module provided by DeepBreath [67] which is able to reconstruct the correct breathing signals of multiple co-located individuals combining an antenna array to Independent Component Analysis (ICA). UWB monitoring methods include device-free or device-based approaches. A technical explanation of this distinction is presented later. The practical consequences are a global better accuracy for device-based UWB, also in real life conditions, i.e., when moving people surround the subject of interest. However, a device-based approach does not generally provide full body monitoring and can suffer from psychological conditioning of the monitored person. In addition, from a clinical point of view, tremor detection will be better performed with a device-based system, while gait and posture monitoring will require a device-free one. However, these powerful adjustments and accuracy standards can further worsen in the singling-out process in real-life conditions, i.e., people moving, if compared to inertial-based devices. A potential drawback is that machine learning models need to be trained since they can “learn” from large-scale datasets. For this purpose, it is worth noting that if the surrounding environment changes, i.e., objects are displaced or furniture in the house is moved to other locations, the accuracy performance lowers, and the model requires to be trained with new datasets. Such intrusiveness in people’s everyday lives also raises confidentiality issues since subjects are pervasively monitored during the day in all rooms and locations of the place of interest. However, the privacy concerns of such a monitoring system seems to be lower compared to the camera-based ones, since this technology is more discrete and tolerable for the subjects. The number, activity and position of monitored people are not observed in their physical appearance but depicted as a variation of signals and subsequent extrapolated data.

### 1.4. Geomagnetic Monitoring

**Figure 3:**
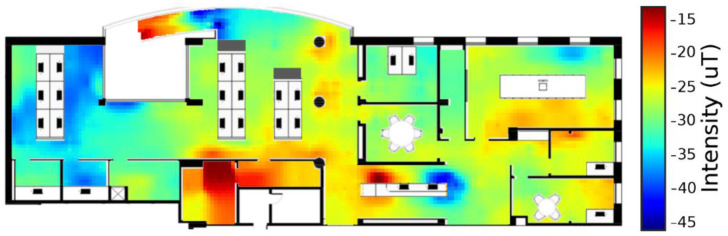
Plan of a building used to test an algorithm for the construction of the magnetic fingerprint. The vertical bar on the right represents the magnetic field intensity. In the floor plan, colour intensity correlates with people activity. Data were collected from six people walking with their smartphones and the walking paths were then acquired (reproduced under the terms and conditions of the Creative Commons Attribution (CC BY) license from [70]).

Among all of the aforementioned signals, the earth magnetic field is a physical medium with promising properties for indoor localization, thanks to its pervasiveness, independence from extra infrastructure and dependence on crowdsourcing [68] (Figure 3). Indoor magnetic fields can achieve good positioning precision at almost no investment and infrastructure-free compared to other technologies [69,70]. Some studies have pointed out the potential outperforming of geomagnetic positioning power compared to traditional RF-based technology in differentiating locations, but have also pointed out the need for real-time and constant location computations with very stable fingerprints and a low accuracy [71]. This has especially been motivated by the increasing power in location estimation due to interference caused by indoor structures. Spatial anomalies in the magnetic field can be detected by a smartphone’s magnetometer and applied as a method of fingerprint indoor localization [68,72]. The practical usage firstly requires the measurement of the geomagnetic signals by the smartphone and the computation of a signal map. Then, localization is established and related signals are processed by a certain algorithm and the corresponding location is returned to the target user [69]. The superiority of geomagnetic-based devices for outdoor monitoring on RF-based technology is overwhelmed by its low positioning resolution standards, in the order of meters, that subsequently limit the application of geomagnetism for fine activities discrimination, such as tremor and falls [73,74,75].

### 1.5. IR-UWB: Physical Basis

Impulse-Radio Ultra-Wideband (IR-UWB) is standardized in IEEE 802.15.4-2015, and is currently the most performant radio positioning technology with centimetre-level accuracy and is used widely in industrial applications [76]. The Federal Communications Commission (FCC) allows the commercial use of Ultra-Wideband (UWB) devices in the frequency band from 3.1 GHz to 10.6 GHz (further divided into individual channels [76] with a very restricted equivalent isotropic radiated power (EIRP) of −41.3 dBm/MHz [77]). The safety issue related to the exposure of humans to the electromagnetic field emitted by the UWB radar is regulated by the guidelines emitted by ICNIRP [78]. This guideline defines the restriction on the basis of the specific absorption rate (SAR), whose values are directly linked to health effects. Research models on the topic rely on UBW signals that satisfy the emission levels issued by the FCC [79]. In the model proposed by Cavagnaro, Pisa [80], a layered body model was considered to evaluate the influence of the different tissues of the human body on the non-ionizing radiation absorption. Results demonstrated that the power actually absorbed from the human body respected the imposed guideline restrictions [80]. Furthermore, the planar inverted cone antenna (PICA) showed the lowest risk of damaging human tissue compared to the other two antennas (CPW-fed inverted cone antenna, and broadband monopolar antenna) with absorption levels below the limits set by FCC [81]. However, the present article does not strictly refer to the standard defined in IEEE 802.15.4-2015 [76], but more generally to any system that employs very short duration and high bandwidth pulses for their communications. It is worth noting the term UWB generally applies to any radio communication system based on a wide bandwidth, typically defined as either a 10 dB bandwidth greater than 20% of the centre frequency or greater than 500 MHz in absolute terms [82]. The methods used for IR-UWB positioning are common for all acoustic and radio wave positioning systems, and involve the collection of location information from radio signals travelling between the node to be localised and a number of reference nodes with known positions [83,84,85] (Table 1).

**Figure 4:**
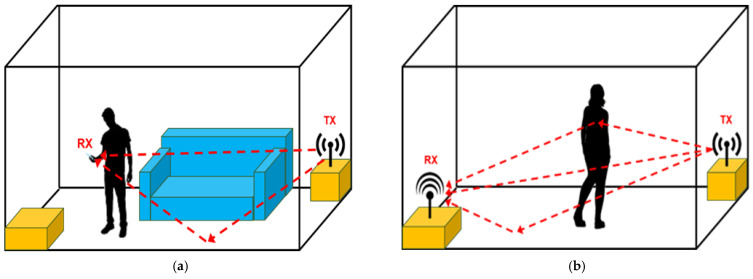
(**a**). Device-based approach. (**b**). Device-free approach. Effect of human presence on multipath environment. An RF signal propagates from the transmitter to the receiver via multiple paths, i.e., multipath components. The presence and position of people furtherly affect the multipath environment, while people motion introduces signal frequency shifts, i.e., Doppler effect.

Signal reflection and diffraction imply the signal received by an antenna is the sum of the attenuated, delayed and possibly overlapping versions of the transmitted signal, i.e., the multipath components. This phenomenon makes localization very challenging, especially in indoor environments, where the NLoS multipath components are predominant. IR-UWB, instead, can provide high ranging and positioning accuracies especially in indoor environments due to its high temporal resolution, low power consumption and multipath immunity [95,96,97]. Moreover, additional mitigation methods have been developed in order to manage the performance degradation problem for NLoS localization, such as the usage of a sparse pseudo-input Gaussian process (SPGP) [98] or the combination of the output of an extended Kalman filter (EKF) and an extended unbiased finite impulse response (EFIR) filter via probabilistic weights [99]. Furthermore, the accuracy of IR-UWB position estimates during strenuous dynamic activities in which moves are characterized by fast changes in direction and velocity has also been evaluated [100]. An in-depth analysis for the selection of the optimal position to mount the UWB sensor for the performance of athletes during training and competition led to a median ranging error of 22 cm [101]. The “standard” UWB positioning methods are “device-based”, since a subject needs to wear one or multiple transceivers that proactively interact with the IR-UWB reference nodes to be localized. In the “device-free” approach, instead, there is no need for people to carry any device. The most traditional device-free localization approach is based on video image processing, but, as previously evidenced, this has several limitations and drawbacks such as being prone to occlusion, insufficient lighting [18], high deployment costs and privacy concerns [102]. This technology is instead based on the fact that the RF propagation channel is continuously modified by the presence of people. As already stated, a transmitted signal propagates to the receiver through multiple paths and each one provides a differently delayed, attenuated, and phase-shifted copy of the transmitted signal. Hence, the received signal results in a combination of several multipath components. The presence and position of people in an environment can deeply affect the multipath environment while their motion can introduce the Doppler effect (see Figure 4a,b). This in turn means that it is possible to analyze RF signals, e.g., Wi-Fi [103] and LTE [49,51], to identify changes produced by the presence of people and their activity [102]. Recently, the device-free approach has also been exploited to perform localization [104,105,106,107], people counting [102,108,109,110,111] and activity recognition [107,112,113,114] via IR-UWB signals. Due to the mentioned characteristics, the measurement and quantification of activity in clinical movement disorders via an IR-UWB radar sensor is also very promising. For example, this new technology has many advantages over traditional tremor detection methods: the UWB system does not require any calibration for tremor amplitude estimation, unlike the video that needs a reference object with a known size in the observed frame. It is worth noting radar systems based on low frequencies, i.e., less than 1 GHz, are not used to detect tremor because of their low temporal and spatial resolution of just a few centimetres [115], which is not adequate for detection of tremors. Moreover, UWB frequencies help to detect still or moving bodies without suffering from interference of other sensors achieving accuracy performances up to 10 cm [43], detecting even fine movements, like breathing. In fact, vital signs monitoring with RF-systems is a non-contact and harmless method to the human body, causing no inconvenience for the patient. Extremely wide transmission bandwidths enhance location accuracy and material penetration, using a large portion of the radio spectrum and low energy levels. High penetrability and non-intrusive standards let the radar sensor invisibly track movements in all indoor environments. This type of movement detection does not require high levels of compliance from the subjects since the radar sensor is lightweight and has no contact to the body. Particularly, they can provide high ranging and positioning accuracies especially in indoor environments [64] and are insensitive to poor lightning conditions [18]. This system can provide a day-long and simultaneous monitoring of a large number of patients in different indoor settings, such as homes, hospitals, and nursing homes. However, the full potential of this indoor monitoring system requires methods and algorithms able respectively to visualize and process the collected data. 

## 2. Clinical Applications

### 2.1. Gait Analysis and Indoor Positioning

Gait analysis is widely used in medicine, and to date the less intrusive technology were based on IMU wearable sensors [87] and camera systems [18,39], however different studies showed that UWB technology could be used for gait analysis. The compact size and versatile functionality of inertial-based devices made them a feasible option for integration with other technical solutions for indoor localization. Some studies investigated the combination of IMU and UWB sensors. The UWB-IMU integrated solution should ideally take advantage of both UWB’s high localization accuracy and IMU’s NLoS localization and motion sensing [43]. Corrales, Candelas [116] used a Kalman filter algorithm to fuse the inertial motion system and the UWB positioning system for motion and position tracking, while Wang and Li [117] integrated IMU and UWB devices for pedestrian positioning and the combined solution and improved the positioning error to about 0.7 m, which is not ideal for many indoor localization applications. Zhang, Zhang [43] proposed a system composed of an accelerometer and gyroscope for human indoor localization and motion tracking. Authors analyzed gait during one-step walking and arbitrary path continuous walking. In the measurement of one-step walking the average errors of the IMU and UWB modules were, respectively, 4.02 cm and 4.70 cm, while the UWB-IMU integrated system improved the accuracy and stability for localization tracking in the tests with rectangular paths. In the tests with an arbitrary path, the UWB-IMU integrated method improved both stability and accuracy of localization performances, in fact the average position error of the combined method was 7.58 cm, which was lower if compared to 11.59 cm and 12.64 cm, achieved by the separate methods. Authors also measured the attitude variation of the foot, simulating foot twisting during walking. The results obtained demonstrated the potentiality of the developed sensor device for real-time gait analysis, measuring the abnormal foot attitude, by the analysis of pitch and roll angles. The authors’ explanation focused on error accumulation of the IMU measurements and the presence random errors that increased when there was a NLoS occlusion between the sensor node and the UWB anchors. Systematic error accumulation reduced the accuracy and required implementation with specific algorithms also to observe the gradient of the UWB location and IMU location with aim of finding barriers and correct the error. Ayena, Chioukh [118] proposed to combine the performances of a radar sensor and a wearable device, such as an instrumented insole, worn at the foot to evaluate the risk of falling during a simple clinical test. Data were recorded during a timed up and go (TUG) test where a stride length (SL) was computed with three different approaches. Each approach was based on an algorithm that calculated SL as a function of the accelerations along the *y*-axis. The authors compared data recorded with insole against the radar outputs and demonstrated that their integration is feasible and gives the possibility to measure dynamics of gait with high accuracy standards. In particular, for a full dataset from a TUG test, the approach based on the algorithm that established a correlation between the maximum and minimum values, average of acceleration and step length algorithm showed a root mean square error (RMSE) equal to 0.3675. Several studies exposed the potential benefits on localization accuracy improvement by integrating radar-based technology and IMU [119,120,121,122]. Best results showed an improvement of location positioning from 3.0 m to 1.5 m with a probabilistic localization algorithm [119] and achieved a localization error of 5.7 m over 5–10 min of indoor walking [120]. 

**Figure 5:**
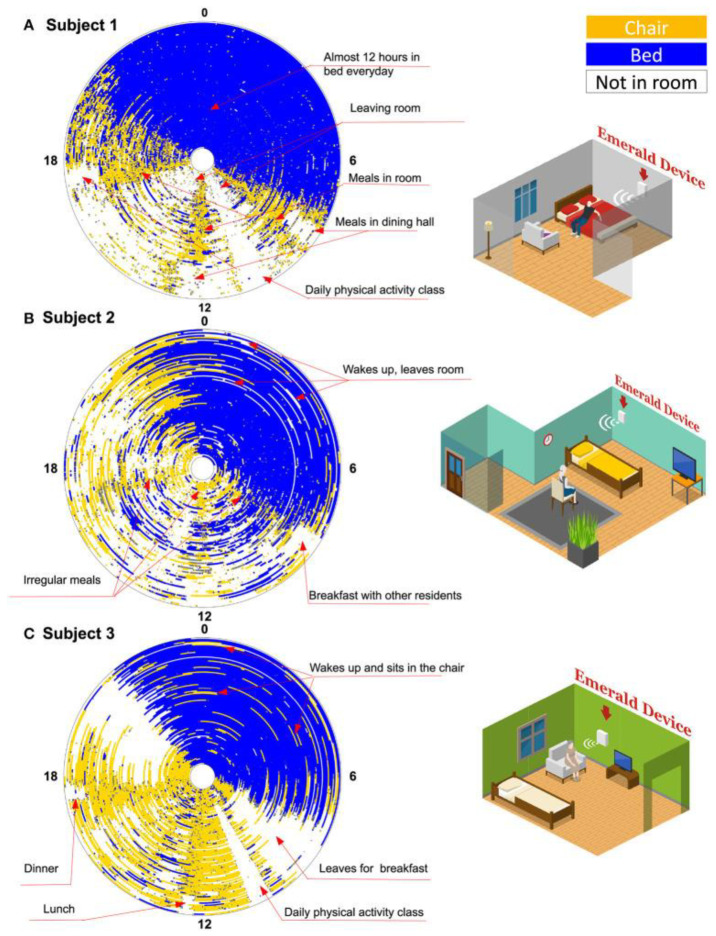
The Emerald RF sensor. A longitudinal view of behavior patterns of three subjects basic activities after becoming COVID negative with different colors referring to different locations inside and outside the room (reproduced under the terms and conditions of the Creative Commons Attribution (CC BY) license from [61]).

IR-UWB technology alone demonstrated promising results for the estimation of foot clearance during walking [123]. Mahfouz, Kuhn [124] tested an UWB 3-D real-time tracking system for an indoor line-of-sight environment with accuracy around 1 cm. Shaban, Abou El-Nasr [64] proposed a low-cost and low-complexity wireless gait tracking system suitable for gait analysis using wearable UWB transceivers, both for indoor and outdoor measurements: the proposed system provided a ranging accuracy of 0.11 cm for the knee-to-ankle distance. In a study by Qi, Soh [125], the authors considered foot clearance, i.e., the vertical distance between a foot and the ground during walking above ground, as a key factor for a better understanding of the complicated relationship between falls and gait. The authors of this study proposed a wearable system based on a pair of small and light UWB antennas placed on a point approximating to the heel/toe of the foot, both in stationary and dynamic conditions. In this way the reflected signal from ground was captured and the distance was estimated via the signal propagation delay to capture both stationary and dynamic measurements. The stationary experiment demonstrated a very good level of adherence between the proposed UWB-based system and a standard motion sensor with correlation coefficient value of 0.9604. Then, the authors allowed the subject to walk forward over 5 metres at a normal speed for 3 times in order to measure foot clearance during gait. In the same study, the authors defined foot clearance during walking as the minimal/maximal vertical distance between the toe/heel and the ground during the swing phase of the gait: the best Heel-to-ground Clearance (MaxHC) and Toe-to-ground Clearance were respectively above 0.15 m and almost 0.2 m. Rana, Dey [126] analyzed human gait with IR-UWB sensing, by the design of a noncontact and non-intrusive wireless gait analysis tool ITERATOR, associated to Kinect Xbox One, that helped to capture 3D human motion and track the skeleton of the human body [127]. The IR-UWB radar transmitted short pulses that enabled the system to be employed in multipath environments. The root mean squared error (RMSE) has been measured between proposed UWB prototype and Kinect sensor results with RMSE resulted of less than 0.5. The proposed UWB-based models demonstrated a non-intrusive, non-contact, wireless way able to recognize physiological gait dynamics and alterations of walking patterns. Different types of walking such as, propulsive, waddling, steppage should be considered for future experiments, as suggested by the authors themselves [126]. The usage of a UWB micro-Doppler radar has been investigated to estimate walking speed [128,129] and to determine a relation between gait velocity and cognitive functions in the elderly [130]. A WiGait-based strategy was designed by Hsu, Liu [131]. This technology is a home sensor hanging on the wall like a picture frame which is able to monitor gait velocity and stride length. WiGait infers a person’s gait by analyzing the surrounding radio signals reflected off his body and does not require him to wear a device. WiGait’s accuracy was between 96.0% and 99.8%, across all subjects for the analysis of gait velocity, while for stride length varied between 88.4% to 99.3%. Subsequently, in the presence of activity-based motion, simulating real conditions (in the presence of desks, chairs and different household items), WiGait’s accuracy in computing gait velocity ranged from 95.3% to 99.8%, while its accuracy in measuring the stride length was between 85.9% to 99.8%. Fan, Li [132] proposed the RF-Diary, an RF-based method that observes and captions in-home daily-life movements both in normal and poor light conditions. To capture objects’ information, besides RF signals, RF-Diary also took in consideration as input the home floormap marked with the size and location of static objects, that provided information about the surrounding environment, enabling the model to infer human interactions with objects. The proposed model obtained comparable results to video-captioning in visible scenarios and continued to work effectively in dark and occluded conditions, where videocaptioning methods failed due to the loss of the line of sight. Integration of the floormaps into the model and the multi-modal feature alignment both contributed significantly to improving performance. In Figure 5 an activity graph of three subjects after becoming COVID negative is presented. This tool can objectively quantify subjects’ daily behaviour, allowing to visualize daily and repetitive activities. In particular, a temporal longitudinal view is shown, as concentric circles represent the days since COVID negativization, from the inner most to the outermost circle, which is the last day of monitoring. Time intervals outside the coverage of the wireless signals are inferred from white patches. Yellow cones reveal the time spent in the subject’s room and the blue ones represent bedtime, while blue circles mean bedtime. A deeper investigation on people’s everyday activities can provide different insights about behavioural phenotyping. For example, the absence of a regular routine could be a sign of subtle agitation and cognitive impairment [61].

Movement disorders have also been tracked via human skeleton pose estimation. RF-Pose is a neural network system that is able to infer 2-D human skeletons by transmitting low-power FMCW (Frequency Modulated Continuous Wave) RF signals and receiving their reflections. Experimental results demonstrated the radio-based system is almost as accurate as the vision-based one used to train it [133]. RF-Pose3D represents an evolution of the previous system which is able to perform 3D pose estimation achieving an average error of 4.2 cm, 4.0 cm, and 4.9 cm along the X, Y, and Z axes respectively [134]. RF-Action represents the first model for skeleton-based action recognition using radio signals in the Wi-Fi range with a mean accuracy above 80% in both visible and occluded scenarios [135]. RF-Avatar leverages the same frequency range for 3D skeleton recovery and is able to achieve in visibility a mean joint position error of 5.84 cm and mean vertex-to-vertex distance of 1.89 cm, while for through-wall scenes and subjects wearing loose costumes the values, respectively, increase to 6.26 cm and to 1.97 cm [136]. 

Another promising indoor monitoring system, is the geomagnetic one. Sun, Wang [137] proposed a fusion indoor positioning method that integrated both the pedestrian dead-reckoning (PDR) and geomagnetic positioning by using the genetic-particle filter (GPF) algorithm. PDR technology uses an accelerometer, gyroscope and magnetometer to obtain continuous positions instead of installing the signal transmitting stations [73,74]. PDR and the geomagnetic positioning were integrated by using the genetic-particle filter (GPF) algorithm: the mean positioning error and the RMSE were, respectively, of 1.72 m and 1.89 m. Moreover, 80% of the test points had a positioning error within 2.45 m. In a study by Chung, Donahoe [75], the authors proposed an indoor positioning system that measured pedestrian location inside buildings and across multiple floors using disturbances of the Earth’s magnetic field caused by structural steel elements in a building and demonstrated accuracy within 1 m 88% of the time in experiments in two buildings and across multiple floors within the buildings. 

**Figure 6:**
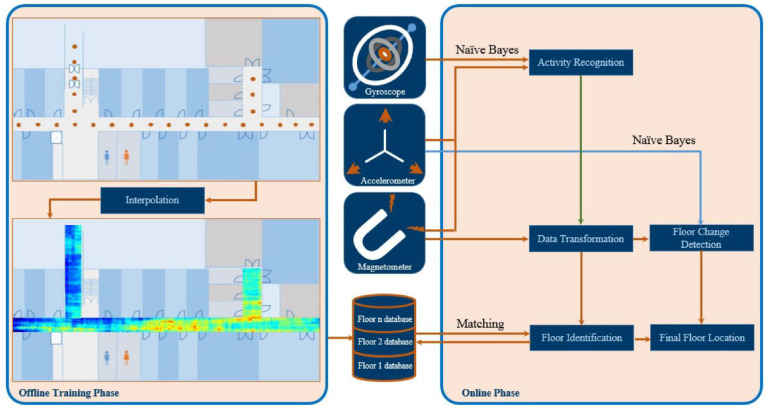
A three-step model for Floor Identification based on magnetic field data captured with smartphone sensors. (reproduced under the terms and conditions of the Creative Commons Attribution (CC BY) license from [5]).

In Figure 6, another model for floor identification based on magnetic fields is depicted. In this three-step model user activity is firstly determined, then, the magnetic field is used for floor identification. In the last step, floor change detection is achieved with the help of accelerometer and magnetometer data [138]. 

### 2.2. Fall Detection

Fall prevention for older patients, is a crucial quality of life improving approach in preventive medicine. Mastorakis and Makris [139] designed a model that recognized the start of a fall considering the exceeding of some thresholds on height and width-depth of 3-D bounding box enclosing the human silhouette. A similar approach, using the 3-D bounding box of the human, was adopted also in [140]. Nghiem, Auvinet [141] detected falls through the vertical speed of the human head and body centroid related to the distance to the ground: the authors obtained the correct classification of 29 falls out of 30. Similarly, Zhang, Liu [142] detected falls by detecting the head region from the human body and the floor level, using a depth reference image. More complex algorithms distinguish falls and other actions in the method proposed in [143], based on the real-time detection of the centre of mass of the moving objects. Then, a statistical model elaborates the probability of a specific action: with a dataset of eight activities, the system achieved a sensitivity of 90% and a specificity of 100% for the falling events. Planinc and Kampel [144] calculated the axes of the head, shoulder centre, spine, hip and knees with the Microsoft Kinect cameras and detected falls when the person was parallel to the ground floor and the distance between spine joint and the ground floor reduced [18]. Panahi and Ghods [38] designed a falling detection method with an image-based SVM algorithm that recognized falls using the distance of the person’s centroid to the floor. They attained sensitivity and specificity of 100% and 97.5%, respectively. Video assessment of movement has found application both for the detection and localization of falls [18,38]. Particularly relevant is the application of the UWB approach to fall monitoring and detection since it offers confidentiality and does not require to wear any instrument [18,41,42]. An approach based on “smart floors” and “sensing floors” has been postulated to record episodes of falls [145], by the analysis of vibrations, pressure, acoustic signals or even occupied surface [145,146]. The use of a UWB technology to detect falls in domestic environments was presented in [147], where the performance of supervised and unsupervised fall detection were compared. The authors applied an unsupervised approach to movement data recorded via an UWB sensor, placed over the ceiling, to detect falls and distinguish them from other types of activities. Two subjects simulated different types of movement activities including falls, normal walk, fast walk and lying. In [148], a convolutional neural network (CNN)-based framework was proposed to classify human actions into “Fall” and “Activities of daily living”. The combination of convolutional layers and convolutional long short term memory (ConvLSTM) demonstrated to be able to extract robust features for fall detection and outperform the CNN-based methods [149]. Diraco, Leone [41] demonstrated the suitability of a radar smart sensor based on IR-UWB sensing and micro-Doppler spectrograms for fall detection: the micro-motion signature and unsupervised learning detected falls with sensitivity and specificity greater than 97% and 90%, respectively. This study showed both the suitability of the radar micro-movement signatures for fall detection and the necessity of a tailored approach for each subject, due to specific fall dynamics. The literature reported for radar sensors an accuracy rate for fall detection variables between approximately 80% up to 100%, and the corresponding false alarm rates between 20% down to close to 0% [18]. Fall detection datasets gave a positive impulse to the diffusion of systems for fall detection: in particular, work was speeded up with the already available data and tested algorithms. Furthermore, a common dataset helped for a comparison of different approaches [24]. 

### 2.3. Tremor Assessment

Accelerometers and gyroscopes have been deployed also for tremor assessment [150]. Tremor frequency error measured with inertial devices is estimated to be less than 0.5 Hz [151]. Over the last years, inertial-based sensors [12,13,14,15,152] aroused increasing attention in the field of fine movement monitoring in the at-home setting or even for objective analysis of tremor. Video assessment of movement has found application for the tremor assessment also with optical tracking systems [153,154,155,156]. In [154], a coloured marker with specific properties of intensity and reflectivity was attached on the tremulous limb and then tremor characteristics were computed by signal-processing techniques of the frames including the tremulous body parts. Analysis of the literature reveals that the tremor frequency estimation obtained by an ordinary video-based system is generally around 0.1 Hz [156]. In particular, in [156], the authors detected tremor frequency using video analysis and the accuracy was compared against the frequency measured by the accelerometer. Results showed that the difference between the frequency from the video and accelerometer was more than 0.2 Hz only in 14% of data. Oikonomidis, Kyriazis [157] proposed a markerless video system for hand tracking. However, markerless techniques were not as accurate as other techniques [158] and the accuracy performance showed an inverse relation with the distance between the camera and the patient. As a result, markerless video systems might not be suitable to give the tracking accuracy needed for tremor characterization. Video systems not only require reflective markers on the subject’s body [159], but are also much more sensitive to variations of lighting in the setting [41]. In addition, several cameras, calibration, and preprocessing techniques are required for a precise construction of each frame. The expensive equipment and the necessity of line-of-sight between the subject and the camera represent further major obstacles that have limited the diffusion of systems based on video detection of movement [160]. The possibility of tracking movement all day long should be the gold standard for the creation of new high-performing video-monitoring systems, since a continuous and pervasive motor assessment could provide, more complete data from a medical point of view, increasing the reliability of the method. Possible privacy concerns may arise from tracking people through walls during the whole day: a challenge-response authentication protocol that prevent people from maliciously using RF signals to see non-authorized areas is feasible [132]. Nowadays, this appears as a mere suspicion with this technology, since framing a moving subject just for an angle of view approximately corresponding to 60 degrees limits the suitability of this system in everyday life. Furthermore, the UWB acquisition system allows a global tremor assessment, i.e., taking into account all tremulous body parts, and can also give information about patient kinematics, which can improve diagnostic accuracy. The work of Blumrosen, Uziel [161] focused on the quantification of tremor using an UWB-based WSN, in which each sensor node was used as a radar to capture tremor during an observation period of 20 s with stationary conditions. The experimental setup included three types of sources of disturbance, such as the presence of metal reflectors, a wooden wall and a person moving his hand with an aluminum foil in the background. The results showed that the sensor node was able to analyze tremor characteristics and the estimation of tremor frequency had an average error of 0.01 Hz. The authors exposed their confidence on the improvement of the performances by increasing the number of sensors, introducing directional antennas, and using higher bandwidth and radiation power. However, the true validity of this system should be tested in more complex contexts, such as environments with thick walls, NLoS conditions, moving people, larger distances between the sensor nodes and the patient, lower transmission power, patients with multiple tremulous body part with different frequencies and amplitudes and small antennas. Blumrosen, Uziel [162] also proposed a technology to assess tremor for the diagnosis of neurological pathologies and its monitoring. A feasibility test was conducted by examining the system performance against an arm model that fluctuated in the range of clinical tremor frequencies (3–12 Hz). The UWB-based acquisition system showed a frequency estimation error of less than 0.1 Hz and provided a set of tremor amplitudes along the tremulous body part. 

### 2.4. Vital Sign Monitoring

**Figure 7:**
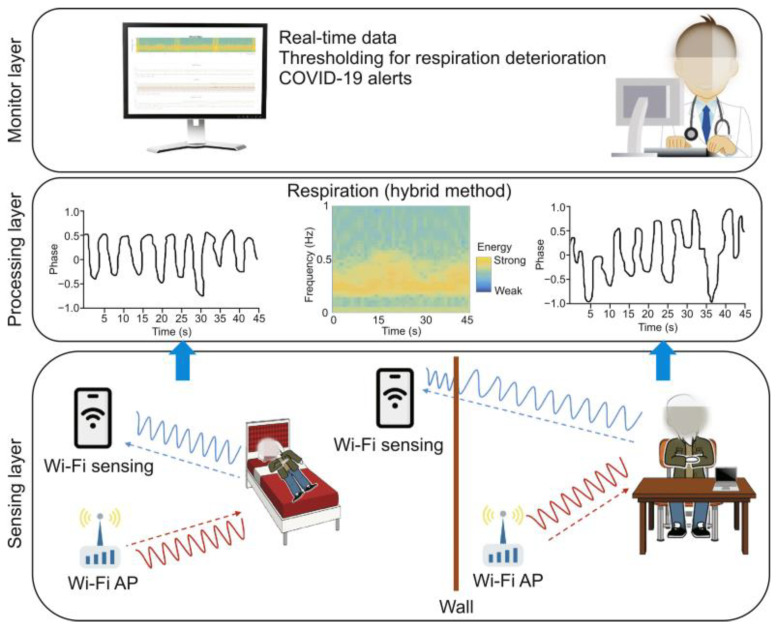
Wi-Fi-based sensing can be used for remote monitoring of human activities during COVID-19 pandemics, such as respiratory dynamics. Three layers summarize the working process of the Wi-COVID from the bottom to the top of the image. First, a Wi-Fi sensing, like Raspberry PI, records the respiratory signal, then, in the processing phase the respiratory parameters are obtained using the Cloud and finally real-time streamed with alerts. (Reproduced under the terms and conditions of the Creative Commons Attribution (CC BY) license from [173,174]).

Since IR-UWB radars have a high resolution, they can be used to detect the fine motion of objects [163]. This means that not only can large movements of the human body but also that small movements, e.g., respiration rate (RR) [164,165,166,167], and heart rate (HR) [165,168] can be detected. The need for contactless respiratory monitoring systems has been further motivated due to the outbreak of the COVID-19 pandemics, as shown in Figure 7. The physical principle for vital signs monitoring is not only based on the evaluation of signal time of arrival but also on changes of frequency and phase after interactions with the subject’s body [169]. IR-UWB and Doppler radars have been proposed for vital sign monitoring [66,163]. These two systems show complementary characteristics, since Doppler radars can estimate velocity but not position, suffering from artifacts due to multiple people, while the IR-UWB technology offers a much higher range of resolution even when multiple people are present. Li and Lin [170] proposed robust methods that require the use of two identical radars for detecting vital parameters in presence of random body movements. Hu and Jin [171] investigated the IR-UWB radar for HR and RR detection with ensemble empirical mode decomposition (EEMD) and continuous wavelet transform (CWT). Radar-based technologies outperformed even photoplestimography for vital sign estimation, especially in contexts with multiple heartbeats, in both accuracy and privacy standards [172]. Diraco, Leone [41] demonstrated the suitability of a radar smart sensor based on IR-UWB sensing and micro-Doppler spectrograms for vital signs monitoring during ADLs in presence of moving subjects in the home setting. Moreover, the authors tested this model for detecting the vital signs during the post-fall phase, by training 30 subjects to simulate falls events. Yue, He [67] proposed a RF-based model to monitor the respiration signals of short-distanced people, also during the night. In particular, this system, known as Deepbreath, used a frequency-modulated carrier waves (FMCW) radio equipped with an antenna array, transmitting a low power wireless signal and capturing its reflections. Subjects were asked to wear a respiration belt, then the signal bounced off the people in the environment and got modulated by their breathing: the reflected signal was used to track the person’s breathing. The complexity of breathing patterns and indoor settings required integration with signal suppression modules. This system was further implemented by a Breathing Separation module that reconstructed the correct breathing signals of multiple co-located individuals. The Motion Detection module, integrated with a convolutional neural network that ignored unpurposeful movements, helped to stabilize breathing signals during movement, while the Identity Matching module assessed the periods of stable breathing with no motion. Authors applied DeepBreath to 13 couples for 21 nights of sleep and a global amount of 151 h of data. The correlation between two breathing belts on the chest and on the belly was around 0.915, revealing a high accuracy of the recovered breathing signals. Specifically, the recovered breathing signal had an average correlation of 0.914 with the breathing belt signal measured on the subject’s chest. DeepBreath also recovered the breathing rates of the monitored subjects with an average error as small as 0.140 breaths per minute. In this study, authors also tested the proposed system with subjects sitting shoulder to shoulder on one couch and obtained an average correlation of 0.922 with the ground truth breathing signals. 

### 2.5. Sleep Monitoring

The medical gold standard for sleep staging is based on Polysomnography conducted overnight at home, hospital or in a sleep lab. Such a polygraphic study requires wearing multiple sensors including an EEG monitor, an EMG monitor, an EOG monitor, ECG monitor, multiple chest bands and nasal probes during sleep [175]. Furthermore, sleep monitoring must deal with many issues, such as poor interference with sleep quality and continuity, privacy preservation and favorable lightning conditions. Accelerometer-based sensors obtained good results [176,177,178], but vision-based systems were more comfortable for the user [179,180,181]. However, privacy-intrusiveness and susceptibility to lightning conditions limited their applicability [179,180,181]. Application of radar-based technology to sleep monitoring is a poorly described issue in literature [182,183,184]. Classical studies in [65] showed poor power to discriminate signals due to sleep from other types of motion. However, analysis of literature shows some studies that focused on sleep staging [185] and apnea detection [186,187]. Yue, Yang [188] tested a RF-based system, known as BodyCompass, to estimate the sleep posture accurately even when the person was still on the bed, by analyzing all reflected waves, including also all the indirect reflections due to multipath. Traditional RF-based models reach up high accuracy performances taking advantage of the motion of the people [133,134]; while, being static asleep in bed is not an ideal scenario to capture fine motion. The authors solved this problem through analysis of the multipath. Results highlighted the high accuracy of BodyCompass during the registration of sleep postures of 26 subjects during more than 200 nights. Accuracy was 94% using one week of data from the user and 83.7% using only 16 min of data. Zhao, Yue [175] studied the role of CNN and recurrent neural network (RNN) in predicting sleep stages, focusing on a new predictive model that learns sleep stages from RF signals. In particular, CNN learns stage-specific features that can distinguish between being awake, rapid eye movements (REM) and from deep and light sleep, while RNN determines if the sleep is light or deep. Once the model was trained without the RNN layer on top of CNN, the overall accuracy decreased by 12.8%. Specifically, the precision to discriminate between light and deep sleep decreased by 23.5%, revealing the fundamental role of RNN. Results also showed that the progression of light and deep sleep through the night was determined by specific temporal patterns [189]. For example, the probability of being in deep sleep decreases as sleep progresses, coherent with the need to go through light sleep before getting into deep sleep. These temporal dynamics of sleep stages can be captured by RNN in order to distinguish light and deep sleep. Hsu, Ahuja [65] proposed a RF-based model, known as EZ-sleep, to assess key sleep parameters: a sensor was attached on the outlet power and identified bed location, bed entries and exits, classified sleep and awake periods and computed sleep parameters by the analysis of reflected waves. The authors measured sleep latency (time between going to bed and falling asleep, SL), sleep efficiency (the percentage of sleep time to the time in bed, or sleep SE), the total sleep time (TST) and the amount of wakefulness after falling asleep (WASO). In this way, they were able to monitor the user via RF signals. Average error in predicting TST, SL, SE, and WASO was 10.3 min, 4.9 min, 2.8%, and 8.2 min, respectively. Then, the study focused on the simultaneous monitoring of multiple subjects sleeping in different beds. Average errors in TST, SL, and WASO were 15.8 min, 7.6 min, 1.8%, 13.1 min, showing acceptable errors and were comparable to the case of a single person [190]. 

## 3. Discussion

**Figure 8:**
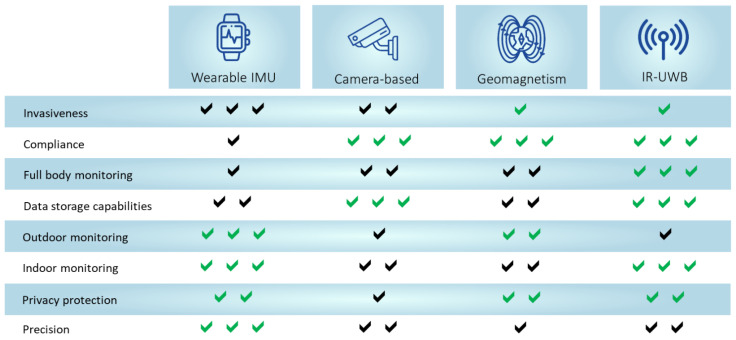
Qualitative features comparison between monitoring systems, in a scale from 1 (**low**) to 3 (**high**).

Recent trends in the field of telemedicine have focused on integrating new technologies and the existing monitoring systems for an objective assessment of the motor performances and vital signs. Movement monitoring can help to recognize both movement disorders themselves but also motor patterns during time spent in the house, while vital signs monitoring can prevent life-threatening events even during sleep. In this context, indoor activities monitoring have drawn attention for several reasons. First, indoor activities appear more difficult than outdoor ones for elderly subjects, since ADLs comprise both gross and fine movements, such as eating, getting dressed and having a bath. In addition, different home settings are dangerous and increase the subjects’ risk of being involved in serious accidents. Furthermore, indoor monitoring accuracy depends so far on several unpredictable factors, such as subject compliance, lightning conditions, physical obstacles, multiple moving people, and privacy concerns. These considerations explain why providing a discrete monitoring ina natural environment is currently a hot topic in the scientific community. In this review, we exposed a deep and comprehensive summary about some of the potential and currently existing methods for patients indoor monitoring and offered a comparison between them (Figure 8).

Traditional inertial devices have good accuracy standards, but they are often uncomfortable, require high standards of compliance and suffer from systematic error accumulation. Optical tracking systems generally need on-body colored markers and line-of-sight for the motor assessment and are affected by privacy concerns. In addition, several cameras, calibration, and preprocessing techniques are required for a precise construction of each frame. Geomagnetism-based technology has high positioning and ranging standards, since it takes advantage of a natural physical medium and does not require any hardware but suffers from positioning resolution standards in the order of meters, that limit its usage for fine movement discrimination. Radiofrequency-based techniques represent a novel and heterogeneous category of indoor localization systems, whose physical principle is based on the analysis of the pattern of reflected waves. IR-UWB is a radar position technology based on a wide bandwidth with high penetrability and not intrusive standards. Thanks to signals excellent penetrability, IR-UWB radars can be installed on the wall and is able to observe the target without attracting any attention from him. Furthermore, radiofrequency -based technology associated with Doppler radars can detect vital signs on the baseline, during ADLs with high precision, since their complementary characteristics help to estimate both velocity and position. This system can be employed in different monitoring settings, such as home, hospital or school to continuously assess movement and detect falls during daily life activities, without requiring high levels of compliance from patients and their caregivers. In addition, IR-UWB guarantees a whole body tremor assessment, with no need of preprocessing algorithms and post-recording filters. Possible benefits could be achieved by the combination of UWB and IMU, such as UWB high accuracy localization standards UWB and IMU localization signal stability, robust NLoS localization and capability for motion tracking. In addition, positioning error and acceptable energy consumption were significantly lower than the conventional methods. However, these studies also pointed out the well-known limit of error accumulation of the IMU measurements, while the UWB measurement showed random errors that increased when there was a NLoS occlusion between the sensor node and the UWB anchors. Systematic error accumulation reduces the accuracy and needs to be implemented with specific algorithms. Furthermore, these devices are bulky, with wired connections, and are uncomfortable for wearable applications [6].

## 4. Conclusions

The aforementioned studies will provide a positive impulse on the use of the radar smart sensors in different real-life scenarios for simultaneous detection of vital signs and critical events [41]. The UWB data transmission capabilities can be further used to transfer data from the sensor node to an UWB hub with internet access to enable long-term monitoring. A hub would then send recorded data to a health care centre for real-time monitoring of vital signs and movement. The goal is a cost-effective, lightweight and energy-efficient contactless indoor localization module based on extremely low radiation that can penetrate walls in any light conditions and collect accurate data continuously. Further studies are still needed to make this technology feasible for clinical practice. However, it is wise to say that clinical practice could take advantage of this novel methodology only in the next future, because RF-technology has only been recently tested in the medical field. It is likely that new trials, involving a larger number of subjects and real-life-simulating situations, will be a needed step. In the short and mid-term, the usage of this technology is far distant since a reliable clinical validation requires a precise standardization with precise protocols. In the future, multiple small size and synchronized UWB-based devices and placed in different locations in an indoor environment are expected to offer continuous non-contact 3-D movement assessment. This would provide a cutting-edge solution in the medical community to recognize acute life-threatening events and prevent them and at the same time diagnose diseases in their early phase. Furthermore, the pervasive monitoring during the entire day is a caveat that highlights the need for privacy issue regulator protocols, preventing possible misuse. A continuous registration of subject’s activities could raise ethical issues, since it could lead to sense human trajectories and locate them in space [191]. However, RF signals are not human-interpretable straightway, since monitored people are depicted as a variation of signals and subsequent extrapolated data. Furthermore, a challenge-response authentication protocol could be introduced to cut down the monitoring for non-authorized areas. New scenarios for remote and automatic monitoring of patients on a regular basis could be finally opened. Long-term radar-based monitoring could be applied to assess new parameters of gait and sleep as opposed to traditional methods, and to establish the progression of disease and the improvements between the clinical visits. Clinical assessment rates would be increased, receiving more information to tailor the therapy based on needs. The ultimate result would be a better control of the symptoms and an easier patient follow-up. 

## Figures and Tables

**Table 1:** IR-UWB positioning system metrics.

IR-UWB Positioning Metric	Definition
Signal Strength (SS)	The target measures the signal strength for received signals from reference nodes in order to use signal strength as an estimator of the distance from them [86]
Time of Arrival (ToA)	The location is the intersection of circles with radius equal to the distance between the target and reference nodes and calculated as the one-way propagation time between them [87,88,89].
Time Difference of Arrival (TDoA)	It is based on comparing the time difference between the target and at least three reference nodes [90,91].
Angle of Arrival (AoA):	The location can be found from the intersection of the angle line for each reference node [92,93,94].
